# ToxBERT: an explainable AI framework for enhancing prediction of adverse drug reactions and structural insights

**DOI:** 10.1016/j.jpha.2025.101387

**Published:** 2025-07-03

**Authors:** Yujie He, Xiang Lv, Wulin Long, Shengqiu Zhai, Menglong Li, Zhining Wen

**Affiliations:** a College of Chemistry, Sichuan University, Chengdu, 610064, China; b Medical Big Data Center, Sichuan University, Chengdu, 610064, China

**Keywords:** Drug safety, Drug-induced adverse drug reactions, Deep learning, Interpretability, Structural alerts

## Abstract

Accurate prediction of drug-induced adverse drug reactions (ADRs) is crucial for drug safety evaluation, as it directly impacts public health and safety. While various models have shown promising results in predicting ADRs, their accuracy still needs improvement. Additionally, many existing models often lack interpretability when linking molecular structures to specific ADRs and frequently rely on manually selected molecular fingerprints, which can introduce bias. To address these challenges, we propose ToxBERT, an efficient transformer encoder model that leverages attention and masking mechanisms for simplified molecular input line entry system (SMILES) representations. Our results demonstrate that ToxBERT achieved area under the receiver operating characteristic curve (AUROC) scores of 0.839, 0.759, and 0.664 for predicting drug-induced QT prolongation (DIQT), rhabdomyolysis, and liver injury, respectively, outperforming previous studies. Furthermore, ToxBERT can identify drug substructures that are closely associated with specific ADRs. These findings indicate that ToxBERT is not only a valuable tool for understanding the mechanisms underlying specific drug-induced ADRs but also for mitigating potential ADRs in the drug discovery pipeline.

## Highlights

• ToxBERT is a transformer-based model that leverages attention and masking mechanisms.
• ToxBERT outperforms existing models in predicting DIQT, DIR, and DILI.
• ToxBERT identifies structural alerts linked to specific ADRs, improving interpretability.
• ToxBERT reduces bias by minimizing reliance on manual molecular fingerprints.

## Graphical abstract

**Figure:**
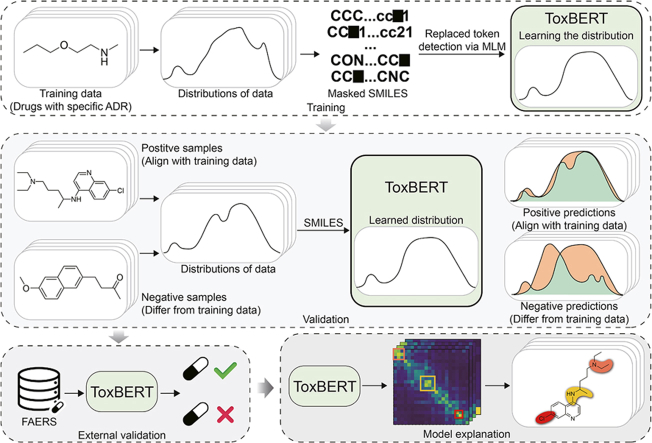

## 1 Introduction

With the continuous development and introduction of new drugs, concerns about drug safety have grown, making its evaluation a critical focus in clinical practice. Despite rigorous pre-market testing, many adverse drug reactions (ADRs) only emerge after widespread use. The unpredictability and severity of drug-induced ADRs, including cardiotoxicity, hepatotoxicity, and other systemic effects [1,2], highlight the limitations of pre-approval clinical trials in fully assessing a drug's safety profile, posing significant safety concerns. Moreover, severe ADRs are one of the primary reasons for drug withdrawals from the market [1]. Even worse, ADRs were estimated to be the fourth to sixth leading cause of death worldwide [3]. Thus, the prevention and early detection of ADRs have become an urgent priority in clinical medicine.

Cohort studies, which track groups of individuals over time, provide a structured framework to understanding the long-term effects of medications on patient health [4]. These studies are particularly valuable for identifying ADRs that may not have been detected during clinical trials. Additionally, spontaneous reporting systems, such as the U.S. Food and Drug Administration (FDA) Adverse Event Reporting System (FAERS) [5], are crucial for monitoring drug safety in real-world settings after a drug's approval [6,7]. Therefore, post-marketing surveillance data play a pivotal role in identifying and managing ADRs, ensuring that unforeseen risks are detected promptly to safeguard patient safety and mitigate public health risks.

With the rapid advancement of machine learning (ML) techniques, ML-based methods are increasingly being applied in pharmacovigilance. ML models excel at analyzing datasets from clinical and post-marketing surveillance, offering faster and more cost-effective approaches to predict ADRs [8]. For instance, a number of drugs can cause prolongation of the QT interval, which refers to the time between the start of the Q wave and the end of the T wave in the electrocardiogram. Drug-induced QT prolongation (DIQT) can lead to severe outcomes, including death. In addition, drug-induced rhabdomyolysis (DIR) and drug-induced liver injury (DILI) are also common and can result in serious consequences. Several studies have successfully employed quantitative structure-activity relationship (QSAR) models [[9], [10], [11], [12]] to predict these ADRs. These advancements underscore the growing role of data-driven methods in enhancing drug safety. In the context of drug-induced ADRs, QSAR models can identify substructures or functional groups, termed structural alerts (SAs), to specific ADRs [13]. However, traditional QSAR approaches often rely heavily on feature engineering, where molecular descriptors are manually selected, introducing potential biases and limitations. While QSAR can indicate whether a compound is likely to be toxic, these models often provide limited mechanistic insights, leaving gaps in understanding which specific substructures are responsible for certain ADRs.

Advancements in deep learning (DL), particularly through the use of simplified molecular input line entry system (SMILES), a textual representation of molecular structures, have significantly improved ADR predictions [14]. SMILES eliminates the need for manual descriptor selection, allowing DL models to process molecules directly as sequential data. Studies have shown that DL models built on SMILES can surpass traditional QSAR models in predicting toxicities [15,16]. The integration of QSAR modeling and DL, referred to as “deep QSAR” [17], has been proposed to enhance model interpretability and accelerate computer-aided drug design. The DeepTox model by Mayr et al. [18] used deep neural networks (DNNs) trained on SMILES data to predict various toxicological outcomes, achieving state-of-the-art results compared to traditional QSAR methods. Winter et al. [19] proposed a model, which translates SMILES to canonical SMILES, enabling the model to effectively learn feature extraction. Models like X-MOL [15], Chemformer [20], and MolFormer [16], which utilize transformer architectures, can be pre-trained on extensive datasets of SMILES representations and capture complex patterns from raw molecular structures. While these models have made significant strides in prediction, the interpretability of their results remains an ongoing challenge. Most DL models for ADR prediction prioritize endpoint accuracy, offering limited insights into which specific substructures within a drug trigger adverse reactions. This interpretability gap is critical because understanding the molecular basis of ADRs is essential for improving drug design and ensuring patient safety. In recent years, interpretability has increasingly been recognized as an essential aspect of artificial intelligence (AI) in drug safety. Recent efforts have focused on applying attention mechanisms and layer-wise relevance propagation to enhance the interpretability of DL models [[21], [22], [23]]. However, the field still lacks a comprehensive integration of explainable AI techniques that connect predictions to molecular substructures, which could significantly improve the practical utility of these models in drug discovery and safety evaluation.

In this study, we present a transformer-based model called ToxBERT, which leverages both attention and masking mechanisms to extract substructures from SMILES and link them to drug-induced ADRs. We validated the model using three specific datasets derived from post-marketing surveillance data: the DIQT atlas (DIQTA), the DIR atlas (DIRA), and the DILI dataset. The model demonstrated significantly superior performance compared to previous studies. More importantly, by employing the attention mechanism, our model highlights the structural patterns associated with specific ADRs, offering a powerful tool to provide deep insights into the molecular mechanisms of drug-induced ADRs.

## 2 Material and methods

### 2.1 Data

#### 2.1.1 Data collection

Three meticulously curated datasets were utilized for model construction and validation in this study, including the DIQTA (https://www.adratlas.com/DIQTA) [9], the DIRA (https://www.adratlas.com) [24], and the DILI dataset (available in the supplementary material of the original publication) [25]. All these datasets were meticulously collected from reliable clinical sources for marketed drugs.

#### 2.1.2 Data preparation

For all three datasets, we retained only organic small molecule drugs while excluding biologics and inorganic drugs.

• DIQTA is a high-quality source of information on drugs with a tendency to induce QT prolongation, categorizing drugs into four concern levels (most-DIQT concern, moderate-DIQT concern, ambiguous, and non-DIQT concern). In DIQTA's schema, ambiguous level drugs demonstrate no QT prolongation in clinical observations, which were excluded. Consequently, 152 positive samples (most-DIQT concern and moderate-DIQT concern) and 99 negative samples (non-DIQT concern drugs), a total of 251 samples were obtained.
• DIRA is a high-quality source of information on drugs with a tendency to induce rhabdomyolysis, categorizing drugs into four concern levels (most-DIR concern, moderate-DIR concern, less-DIR concern, and none-DIQT concern). Ultimately, 154 positive samples (most-DIR concern, moderate-DIR concern, and less-DIR concern) and 39 negative samples (none-DIQT concern), a total of 193 samples were obtained.
• DILI is a high-quality source of information on drugs with a tendency to induce liver injury, categorizing drugs into two types. Ultimately, 599 positive samples (inducing liver injury) and 392 negative samples (not inducing liver injury), a total of 991 samples were obtained.

To validate the robustness of our model, we randomly partitioned the datasets five times, each with a unique random seed. Specifically, positive samples were divided into training, validation, and test sets with a ratio of 8:1:1. Since negative samples were not utilized during training, they were evenly distributed between the validation and test sets with a ratio of 1:1. To address potential class imbalance in the test set resulting from our training strategy, we applied random down-sampling to the negative samples after partitioning. For DIQTA, half of the negative samples were retained; DIRA, all negative samples were kept; and for DILI, one-third of the negative samples were selected to ensure class balance.

#### 2.1.3 SMILES and tokenization

SMILES provides a character string representation of a molecule through a depth-first pre-order traversal of its molecular graph [26]. SMILES efficiently encodes atoms, bonds, and cycles as textual representations, enabling the application of natural language processing techniques to molecules. Given the limited dataset size, we applied SMILES enumeration [27] to augment the training data.

SMILES were retrieved from DrugBank [28] using DrugBank IDs. We obtained canonical SMILES and implemented SMILES enumeration using the RDKit package in Python. The Hugging Face tokenizer was applied to tokenize the SMILES, and we modified the vocabulary table from MolFormer's [16] by reducing its size from 2357 tokens (including 5 special tokens) to 56 tokens (with 5 special tokens). Further details are provided in Section S1 in the Supplementary data.

### 2.2 Model

#### 2.2.1 Attention

Transformer models offer a scalable, flexible, and efficient framework for modeling sequence data. They also have become essential tools across various domains, including drug discovery and molecular property prediction [16,29]. The foundation of the transformer is self-attention, also known as scaled dot-product attention, which effectively models internal relationships within the input by capturing interactions between tokens. This mechanism enables the model to selectively focus on relevant parts of the input data and learn complex relationships within sequential data. In molecular applications, self-attention empowers the model to effectively capture intricate relationships between atoms or molecular substructures.

(1) $$\begin{array}{c} \text{Attention}\left(Q,K,V\right)=\text{softmax}\left(\frac{QK^{T}}{\sqrt{d_{k}}}\right)V \end{array}$$

Where Q, K, and V represent the query, key, and value matrices, respectively, and $d_{k}$ is the dimensionality of the keys. This allows the model to compute scores between queries and keys, facilitating the extraction of relevant information across tokens.

Additionally, multi-head attention enhances this capability by dividing the attention process into multiple heads. Each head performs self-attention independently, attending to different features and capturing richer and more diverse aspects of the data.

(2) $$\begin{array}{c} \text{MultiHead}\left(Q,K,V\right)=\text{Concat}\left(\text{hea}d_{1},...,\text{hea}d_{h}\right)W^{O} \end{array}$$

(3) $$\begin{array}{c} \text{where}\;\text{hea}d_{i}=\text{Attention}\left(QW_{i}^{Q},KW_{i}^{K},VW_{i}^{V}\right) \end{array}$$

#### 2.2.2 Masked language model (MLM)

The MLM was introduced by bidirectional encoder representations from transformers (BERT) [30]. In MLM, certain portions of the input are randomly masked, and the model's objective is to predict the original content of the masked parts using only the context. MLM is frequently used for pre-training, as it enables models to learn information and features across sequences, thereby enhancing the understanding of relationships within sequences. Moreover, MLM has been widely adopted as an effective method for learning molecular features [14].

#### 2.2.3 Efficiently learning an encoder that classifies token replacements accurately (ELECTRA)

In this work, we utilized the architecture and training method of the ELECTRA [31] model. Modifications were introduced to tailor the model for our specific task, as discussed in detail in the following section.

ELECTRA improves upon BERT [30] by introducing a more sample-efficient task called replaced token detection, the objective of which is to distinguish between “original” input tokens and “replaced” tokens generated by a separate neural network, analogous to generative adversarial networks [32]. Specifically, ELECTRA contains two models, a generator and a discriminator. The generator is trained as an MLM, replacing the masked tokens, while the discriminator is trained to identify which tokens are replaced by the generator. This approach enables the model to learn more effectively with fewer computational resources and addresses the token mismatch issue between training and inference in BERT, ensuring consistency of inputs across both phases.

### 2.3 Model construction

Recognizing that DL is fundamentally about fitting data distributions and capturing intricate patterns within training data, we noted that when there is a significant difference between the distributions of two types of samples, a model trained exclusively on single-type samples would struggle to generalize to the distribution of another type.

To address this, we proposed ToxBERT, which leverages the attention mechanism, the masking mechanism, and the ELECTRA architecture. The attention mechanism allows the model to capture intricate relationships between atoms and substructures, while the masking mechanism enables the model to learn underlying representations of drugs. The ELECTRA architecture, particularly the replaced token detection task, plays a crucial role in helping the model learn to identify subtle variations among samples. As a result, ToxBERT can be trained on single-type samples and effectively distinguish two different types of samples.

Python and Bash code for ToxBERT is available at GitHub (https://github.com/FEIFEIEIAr/ToxBERT).

#### 2.3.1 Training

We essentially followed the training method of ELECTRA. The primary difference is that only single-type samples were used for training. Specifically, only drugs with specific ADRs were included in the training process.

**Fig. 1:**
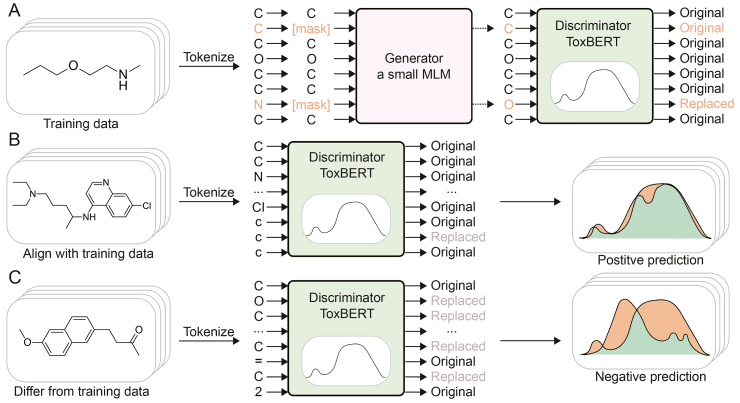
Training and inference process of ToxBERT. (A) Training process of ToxBERT. (B) Inference process for positive sample. (C) Inference process for negative sample. MLM: masked language model.

As shown in Fig. 1A, during the training process, we randomly masked SMILES tokens. The generator was then trained to replace these masked tokens with new tokens, while the discriminator was tasked with distinguishing each token as either original or replaced by comparing it to the original SMILES. This method facilitated effective learning, even with a small dataset, by maximizing the utilization of each token. After training, the discriminator could fit the distribution and recognize patterns in the training samples. Since the discriminator focused on detecting replaced tokens, it became highly sensitive to discrepancies or variations in molecular structures.

#### 2.3.2 Inference

The main difference between our model and ELECTRA lies in the inference stage, where we adapt the discriminator's output to perform a token-level comparison for classification.

As shown in Figs. 1B and C, during inference, we evaluated each token in the SMILES sequence by thresholding the discriminator's output to determine whether it was original or replaced. This threshold could be adjusted as a hyperparameter or set to a fixed value based on task requirements. We set it equal to the masking ratio. If the majority of tokens are identified as original, this indicates that this drug's distribution and structure align with the training data, suggesting it likely belongs to the same type. Conversely, if a significant portion of the tokens are identified as replaced, it indicates that this drug's distribution and structure differ from the training data, suggesting it likely belongs to a different type.

(4) $$\begin{array}{r} \text{Probability}=\frac{\text{Number}\;\text{of}\;\text{replaced}\;\text{tokens}}{\text{Molecular}\;\text{length}}\times 100\% \end{array}$$

This probability represents the proportion of replaced tokens. A threshold is applied to this probability to determine whether a drug is classified as different from the training data. This threshold is adjustable and can serve as a tunable hyperparameter for further optimization. Lower thresholds could lead to higher sensitivity in detecting structural differences within the drug, identifying subtle deviations from the training data. On the other hand, higher thresholds allow greater tolerance for minor variations, focusing on more substantial differences. In our work, we used Youden's J statistic (Youden's index) [33] to determine this threshold. Youden's index helps balance the false positives and false negatives by selecting the threshold that maximizes the index. The index is calculated as follows:

(5) $$\begin{array}{c} J=sensitivity+specificity-1 \end{array}$$

(6) $$\begin{array}{c} =\frac{\text{TP}}{\text{TP}+\text{FN}}+\frac{\text{TN}}{\text{TN}+\text{FP}}-1 \end{array}$$

### 2.4 Evaluation of model performance

#### 2.4.1 Evaluation with classification metrics

To evaluate the performance of the model, we used nine performance metrics in this study, including accuracy, recall rate, precision, Matthew's correlation coefficient (MCC), balanced accuracy score (BACC), F1 score, the area under the receiver operating characteristic curve (AUROC), the area under the precision-recall curve (AUPRC) and specificity.

(7) $$\begin{array}{c} \text{Accuracy}=\frac{\text{TP}+\text{TN}}{\text{TP}+\text{TN}+\text{FP}+\text{FN}} \end{array}$$

(8) $$\begin{array}{c} \text{Recall}\;\text{rate}=\frac{\text{TP}}{\text{TP}+\text{FN}} \end{array}$$

(9) $$\begin{array}{c} \text{Precision}=\frac{\text{TP}}{\text{TP}+\text{FP}} \end{array}$$

(10) $$\begin{array}{c} \text{MCC}=\frac{\text{TP}\times \text{TN}-\text{FP}\times \text{FN}}{\sqrt{\left(\text{TP}+\text{FP}\right)\left(\text{TP}+\text{FN}\right)\left(\text{TN}+\text{FP}\right)\left(\text{TN}+\text{FN}\right)}} \end{array}$$

(11) $$\begin{array}{c} \text{BACC}=\frac{\text{TPR}+\text{TNR}}{2} \end{array}$$

(12) $$\begin{array}{c} F1\;\text{score}=\frac{2\times \left(\text{precision}\times \text{recall}\;\text{rate}\right)}{\text{precision}} \end{array}$$

(13) $$\begin{array}{c} \text{AUROC}=\int_{x=0}^{1}\text{TPR}\left(\text{FP}R^{-1}\left(x\right)\right)dx \end{array}$$

(14) $$\begin{array}{c} \text{AUPRC}=\int_{-\infty }^{+\infty }\text{precision}\left(dP\left[Y\leq x\right]\right) \end{array}$$

(15) $$\begin{array}{c} \text{Specificity}=\frac{\text{TN}}{\text{TN}+\text{FP}} \end{array}$$

#### 2.4.2 Evaluation with external data

The proportional reporting ratio (PRR) is a widely used measure to identify drug-ADR associations in pharmacovigilance [[34], [35], [36]]. The PRR is defined as the ratio between the frequency of reports of a specific ADR associated with a target drug and the frequency of the same ADR reported for all other drugs. PRR and its confidence interval are calculated as follows:

(16) $$\begin{array}{c} \text{PRR}=\frac{N_{TT}/N_{AT}}{N_{TA}/N_{AA}} \end{array}$$

(17) $$\begin{array}{c} \text{PRR}\;\left(95\%\;\text{confidence}\;\text{interval}\right)=e^{\ln\left(\text{PRR}\right)\pm 1.96\sqrt{\frac{1}{N_{TT}}-\frac{1}{N_{AT}}+\frac{1}{N_{TA}}-\frac{1}{N_{AA}}}} \end{array}$$

In these equations, $N_{TT}$ represents the number of reports of the target ADR for the target drug, and $N_{AT}$ represents the total number of ADRs reported for the target drug. Similarly, $N_{TA}$ represents the number of reports of the target ADR for all other drugs, and $N_{AA}$ represents the total number of ADRs reported for all other drugs.

A higher PRR generally suggests a stronger likelihood that a drug is associated with a specific ADR. Therefore, drugs with higher PRRs may be more likely to be predicted as drugs with specific ADRs, while those with lower PRRs may be more difficult to distinguish. PRR was utilized to evaluate our model's performance.

We respectively extracted PRRs for drugs associated with QT prolongation, rhabdomyolysis, and liver injury from the FAERS Quarterly Data Extract Files (Q1 2004 to Q1 2023) [5]. Duplicates, biologics, and inorganic drugs were excluded. Then overlapping drugs present in curated datasets were excluded from external data based on canonical SMILES, respectively. Additionally, considering that 1 is the lower limit of the 95% confidence interval and serves as a detection criterion for ADRs [36], drugs with a PRR less than 1 were removed.

We then ranked drugs by PRR in descending order, incrementally adding them to the test set in groups of ten and calculating the recall rate at each step.

## 3 Result

### 3.1 Model performance in predicting drug-induced ADRs

#### 3.1.1 Model performance on DIQTA, DIRA, and DILI datasets

Three meticulously curated datasets, namely DIQTA, DIRA, and DILI, were employed to assess the performance of our model, ToxBERT. Our model utilized attention and masking mechanisms and was designed to train with single-type data to predict ADRs induced by marketed drugs. A total of nine performance metrics were utilized to evaluate our model. The results achieved in predicting the risk of DIQT, rhabdomyolysis, and liver injury were listed in Table 1. Our model achieved high AUROC across three datasets, indicating satisfactory predictive performance for these ADRs. The average recall rate exceeded 0.9 for DIQTA, 0.8 for DIRA, and 0.7 for DILI, demonstrating its ability to accurately identify drugs with specific ADRs. However, the specificity was slightly lower, indicating a relatively high false-positive rate. We randomly partitioned the datasets five times, and obtained small standard deviations across all metrics.

**Table 1:** Performance of our model on three datasets.

Datasets	AUROC	F1	Accuracy	AUPRC	BACC	MCC	Precision	Recall	Specificity
DIQTA	0.839 ± 0.015	0.766 ± 0.014	0.783 ± 0.018	0.945 ± 0.009	0.806 ± 0.014	0.604 ± 0.028	0.664 ± 0.034	0.913 ± 0.064	0.700 ± 0.061
DIRA	0.759 ± 0.014	0.708 ± 0.010	0.717 ± 0.021	0.763 ± 0.030	0.718 ± 0.018	0.444 ± 0.030	0.660 ± 0.035	0.800 ± 0.025	0.650 ± 0.063
DILI	0.664 ± 0.004	0.658 ± 0.036	0.651 ± 0.023	0.860 ± 0.012	0.658 ± 0.012	0.311 ± 0.053	0.621 ± 0.026	0.725 ± 0.073	0.584 ± 0.047

AUROC: area under the receiver operating characteristic curve; AUPRC: area under the precision-recall curve; BACC: balanced accuracy score; MCC: Matthew's correlation coefficient; DIQTA: drug-induced QT prolongation atlas; DIRA: drug-induced rhabdomyolysis atlas; DILI: drug-induced liver injury.

The aforementioned results are based on random splitting, but this approach may have some limitations. To address this, we also explored the impact of scaffold splitting on the model's performance in Section S2 in the Supplementary data. As shown in Fig. S1, performance differences between random and scaffold splitting were minimal.

#### 3.1.2 Comparison with previous QSAR methods

We compared the performance of our model with previously reported methods. MolFormer-XL-CNN [37], which utilized the pre-trained MolFormer model, achieved the highest AUROC on DIQTA and a competitive result in DIRA. Long et al. [10] achieved the best performance among ML methods in DIQTA using a support vector machine. Zhou et al. [12] obtained the best result in DIRA using a random forest. Li et al. [25] trained DeepDILI with different molecular fingerprints and achieved the best results in DILI.

**Fig. 2:**
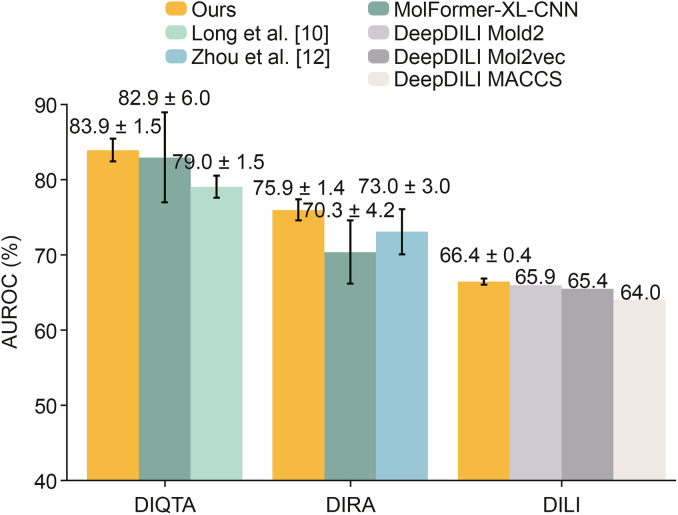
Comparison of our model's performance with previous studies across three datasets. The Y-axis represents the area under the receiver operating characteristic curve (AUROC) achieved by models. The X-axis represents the various datasets. DIQTA: drug-induced QT prolongation atlas; DIRA: drug-induced rhabdomyolysis atlas; DILI: drug-induced liver injury; XL: extra-large; CNN: convolution neural network; MACCS: molecular access systems.

As shown in Fig. 2, our model achieved the highest AUROC scores across all three datasets compared to these models [10,12,25,37]. Additionally, the standard deviations of our model were consistently lower than those of other methods.

#### 3.1.3 Variations of performance with hyperparameters

We utilized DIQTA as an example to analyze the impact of several hyperparameters on model performance.

##### 3.1.3.1 Masking ratio

Our model utilized the masking mechanism to learn contextual information, enabling it to effectively capture drug features. Therefore, we investigated the impact of the masking ratio on the model's performance.

**Fig. 3:**
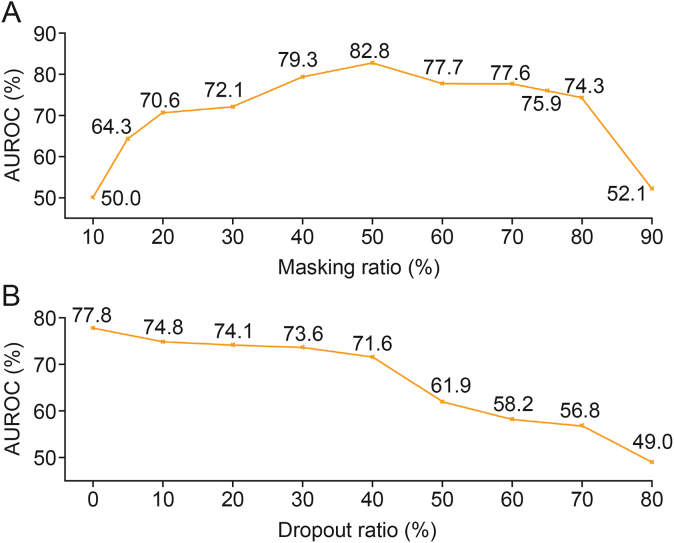
Performance variations with hyperparameters. (A) The impact of masking ratio on model performance. The Y-axis represents the area under the receiver operating characteristic curve (AUROC) achieved by models. The X-axis represents the various masking ratios used for training the models. (B) The impact of dropout ratio on model performance. The Y-axis represents the AUROC achieved by models. The X-axis represents the various dropout ratio used for training the models.

Fig. 3A shows the influence of masking ratio. Optimal masking ratios were broad, with values between 40% and 70% achieving plausible results. Our model struggled to capture features effectively when the masking ratio was 15%, which contrasts with BERT's typical masking ratio of 15% [30]. When masking ratios were below 20% or above 80%, the model's performance rapidly deteriorated.

##### 3.1.3.2 Dropout ratio

In DL, dropout [38] is a widely used regularization technique to prevent overfitting by randomly setting neuron outputs to zero during training. Dropout can introduce randomness into models, thus enhancing robustness and reducing overfitting.

Based on our model and task design, we aimed to leverage certain aspects of overfitting, so we investigated the effect of dropout on model performance. As shown in Fig. 3B, our model's performance decreased as the dropout rate increased, and it struggled to learn effectively when the dropout rate exceeded 50%, which was in contract to other models as expected.

Analyses of additional DL techniques related to overfitting are provided in Section S3 in the Supplementary data. As shown in Figs. S2 and S3, the model's performance is influenced by those techniques.

### 3.2 External validation

#### 3.2.1 Model performance on external data

**Fig. 4:**
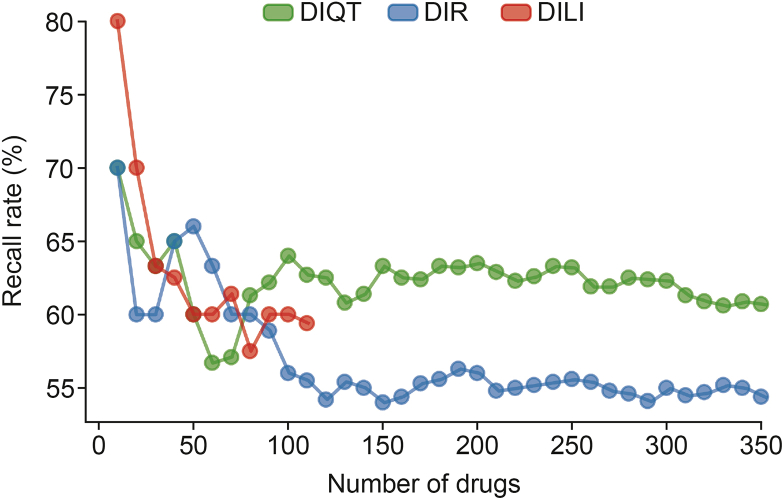
Trends in recall rates with increasing number of drugs included in prediction. The Y-axis indicates the recall achieved by our model. The X-axis represents the number of drugs used for prediction, which were incrementally added based on their proportional reporting ratio (PRR) values. DIQT: drug-induced QT prolongation; DIR: drug-induced rhabdomyolysis; DILI: drug-induced liver injury.

We identified 346,344, and 101 drugs with PRRs for DIQT, DIR, and DILI, respectively, from FAERS. Considering that AUROC effectively reflects the model's classification capability, we selected the model with the highest AUROC for each type of ADR to perform external validation. As shown in Fig. 4, as more drugs with reduced PRR values were included, recall rates generally decreased due to the lower risks associated with these drugs. DIR exhibited the steepest and most pronounced decline. However, our model maintained high prediction accuracy for drugs with high PRR values.

In Table 2, we present the prediction results for the top 10 drugs with the highest PRRs. For both DIQT and DIR, 7 out of the top 10 drugs (70.0%) were correctly predicted by our model. For DILI, 8 out of the top 10 drugs (80.0%) were correctly predicted.

**Table 2:** Top 10 drugs with the most severity of three adverse drug reactions (ADRs) in U.S. Food and Drug Administration (FDA) Adverse Event Reporting System (FAERS) dataset.

ADRs	Generic/proper name	PRR	ATC code
DIQT	Lactulose	14.262	A06
	Aminocaproic acid	12.526	B02
	Felbamate^a^	12.526	N03
	Ripretinib^a^	10.220	L01
	Imatinib^a^	10.214	L01
	Asciminib^a^	10.214	L01
	Omacetaxine mepesuccinate	9.875	L01
	Edaravone^a^	9.122	N07
	Isocarboxazid^a^	6.699	N06
	Amphotericin b^a^	6.150	G01, A07, J02, and A01
DIR	Betaine^a^	51.297	A09 and A16
	Maribavir^a^	17.265	J05
	Trifluoperazine^a^	11.266	N05
	Dexamethasone	11.166	C05, S02, D07, R01, D10, H02, S01, S03, and A01
	Valproic acid^a^	10.122	N03
	Pralatrexate^a^	9.855	L01
	Quazepam^a^	9.849	N05
	Sugammadex	8.089	V03
	Ibuprofen^a^	8.089	C01, M02, N02, M01, G02, and R02
	Rocuronium	8.089	M03
DILI	Vancomycin	6.188	S01, A07, and J01
	Lacosamide^a^	6.188	N03
	Isosulfan blue^a^	4.763	–
	Semaglutide	4.442	A10
	Abrocitinib^a^	4.309	D11
	Maribavir^a^	3.807	J05
	Prednicarbate^a^	3.702	D07
	Crotamiton^a^	3.690	–
	Desonide^a^	3.482	S01 and D07
	Sofosbuvir^a^	3.444	J05

–: no data; PRR: proportional reporting ratio; ATC: anatomical therapeutic chemical; DIQT: drug-induced QT prolongation; DIR: drug-induced rhabdomyolysis; DILI: drug-induced liver injury.

a Drugs that were predicted as positives by our model.

#### 3.2.2 Model performance on different therapeutic categories

We analyzed the Anatomical Therapeutic Chemical (ATC) codes for drugs extracted from FAERS. Distributions of these three types of drugs across different therapeutic categories are shown in Table S1. For all three types, antineoplastic and immunomodulating agents had the largest number of drugs, while the antiparasitic products, insecticides, and repellents had the smallest.

**Fig. 5:**
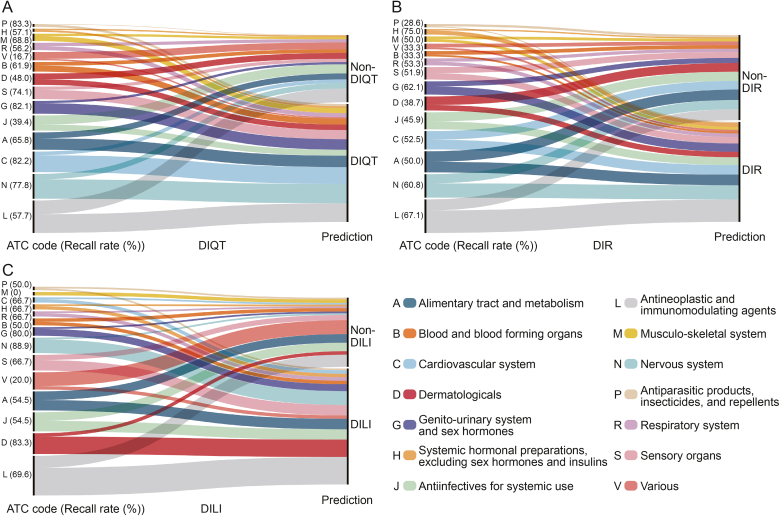
Recall rates achieved by our model across various therapeutic categories using U.S. Food and Drug Administration (FDA) Adverse Event Reporting System (FAERS) data for external validation. (A) Recall rates for predicting drug-induced QT prolongation (DIQT) drugs. (B) Recall rates for predicting drug-induced rhabdomyolysis (DIR) drugs. (C) Recall rates for predicting drug-induced liver injury (DILI) drugs. P: antiparasitic products, insecticides, and repellents; H: systemic hormonal preparations, excluding sex hormones and insulins; M: musculo-skeletal system; R: respiratory system; V: various; B: blood and blood forming organs; D: dermatologicals; S: sensory organs; G: genito-urinary system and sex hormones; J: antiinfectives for systemic use; A: alimentary tract and metabolism; C: cardiovascular system; N: nervous system; L: antineoplastic and immunomodulating agents; ATC: anatomical therapeutic chemical.

We also evaluated the recall rates of our model for each therapeutic category, as shown in Fig. 5. For DIQT, the top three therapeutic categories based on recall rates were antiparasitic products, insecticides, and repellents (P), cardiovascular system (C), and genito-urinary system and sex hormones (G), with recall rates of 83.3% (5/6), 82.2% (37/45), and 82.1% (23/28), respectively. For DIR, the top three categories were systemic hormonal preparations, excluding sex hormones and insulins (H), antineoplastic and immunomodulating agents (L), and genito-urinary system and sex hormones (G), with recall rates of 75.0% (9/12), 67.1% (49/73), and 62.1% (18/29), respectively. For DILI, the top three categories were nervous system (N), dermatologicals (D), and genito-urinary system and sex hormones (G), with recall rates of 88.9% (8/9), 83.3% (10/12), and 80.0% (4/5), respectively. Our model is particularly sensitive to drugs with specific ADRs within these categories, excelling in predictions for the genito-urinary system and sex hormones across all three types.

Considering that the association between a drug and an ADR is highly complex, we have provided a detailed analysis of the potential impact of factors such as age and sex on detection signals. As Table S2 shows, even when influenced by these factors, the PRR-based results in this study remain highly reliable. Detailed information is provided in Section S4 in the Supplementary data.

### 3.3 SAs highlighted by attention mechanism

We validated our model's capability on both curated datasets and external data, demonstrating its ability to extract meaningful drug features. Building on this, we conducted an in-depth exploration of the relationship between ADRs and molecular substructures. All analyses were conducted using the models with the highest AUROC for each ADR.

#### 3.3.1 Insights into embedded features using t-distributed stochastic neighbor embedding (t-SNE) projection

**Fig. 6:**
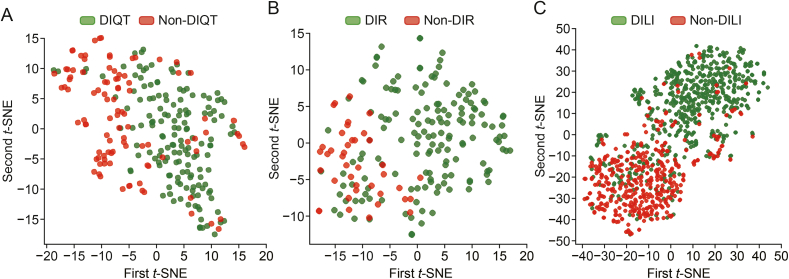
The *t*-distributed stochastic neighbor embedding (*t*-SNE) projection of embedded vectors for three datasets: *t*-SNE projections for drug-induced QT prolongation (DIQT) drugs (A), drug-induced rhabdomyolysis (DIR) drugs (B), and drug-induced liver injury (DILI) drugs (C).

We investigated *t*-SNE [39] to project embedded vectors from the layer before the output, assessing whether our model captures drug features. As shown in Fig. 6, the projections of embedded vectors were clustered into two groups.

For DIQTA (Fig. 6A), the clustering of two types of data was apparent, with a clear boundary separating them. Although some overlap existed between DIQT drugs and non-DIQT drugs, it primarily occurred at the edges of the clusters. This suggests that while both types of drugs may share certain structural features, the model can distinguish significant differences, particularly among drugs located at each cluster's center.

For DIRA (Fig. 6B), clustering of rhabdomyolysis-inducing drugs versus those without specific ADRs was evident. However, the boundaries were less distinct compared to DIQTA and DILI. The overlap between DIR and non-DIR drugs was more prominent, with non-DIR drugs blending more into the DIR cluster. Despite this overlap, the model still demonstrated the ability to differentiate these classes, capturing important structural distinctions.

For DILI dataset (Fig. 6C), the clustering between drugs with and without liver injury was also distinct, though the overlap between these categories was more pronounced compared to DIQTA. The overlap indicates more shared features between DILI and non-DILI drugs, but our model still successfully identified key differences, as those overlapping non-DILI drugs positioned closer to the clusters’ periphery.

#### 3.3.2 Highlighting SAs in coronavirus disease 2019 (COVID-19) drugs related to DIQT risk

Two aminoquinoline drugs, chloroquine and hydroxychloroquine, are approved for the treatment and prophylaxis of malaria at high recommended dosages. They were used globally as a potential, albeit unproven, treatment option for COVID-19 in recent years. There is evidence that chloroquine and hydroxychloroquine may induce significant QT-interval prolongation and increase the risk of arrhythmia, potentially putting patients at risk for torsades de pointes and sudden cardiac death [[40], [41], [42]].

**Fig. 7:**
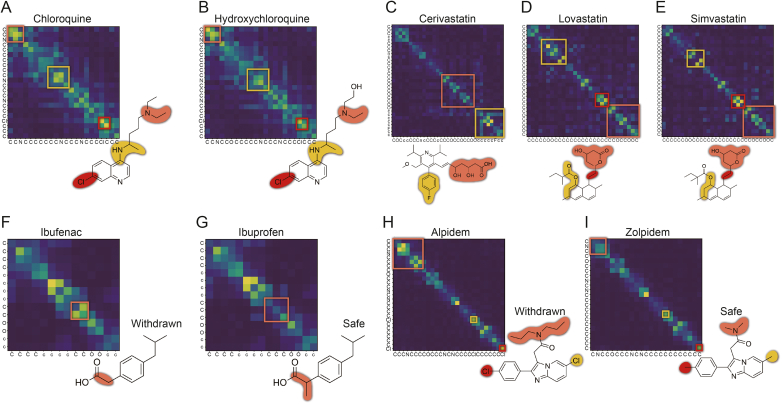
Attention map analysis and highlighted structural alerts (SAs). The sub-figures showed the highlighted SAs and corresponding areas in attention map for chloroquine (A), hydroxychloroquine (B), cerivastatin (C), lovastatin (D), simvastatin (E), ibufenac (withdrawn) (F), ibuprofen (G), alpidem (withdrawn) (H), and zolpidem (I).

Given this context, we investigated the relationship between ADRs and molecular structures by inspecting the attention maps of chloroquine and hydroxychloroquine to explore the information embedded in their molecular structures, as shown in Figs. 7A and B.

Given their structural similarity, the attention maps of chloroquine and hydroxychloroquine closely aligned, highlighting identical groups. Additionally, we identified three key substructures in the attention maps. Subsequently, we analyzed these SAs, referencing previous work [10], which investigated SA distribution variations between QT-prolonging and non-QT-prolonging drugs by quantifying the proportion of each SA. The differences were then calculated by subtracting the proportion in non-QT from the proportion in QT. Notably, several SAs were identified in this previous work, including tertiary amines, sp3-hybridized carbon atoms, and aryl halides, which exhibit significant differences between QT and non-QT drugs, with differences of 48.5%, 43.4%, and 23.8%, respectively, as shown in Table 3. Our model, designed to distinguish differences between QT-prolonging and non-QT-prolonging drugs, focused on structures that closely matched with those identified in previous work.

**Table 3:** Identified structural alerts (SAs) and distribution variations between QT-prolonging and non-QT-prolonging drugs.

Name	SAs	Proportion in QT (%)	Proportion in non-QT (%)	Difference (%)
Tertiary amines		61.1	12.6	48.5
sp3-hybridized carbon atoms		81.3	37.9	43.4
Aryl halide		37.5	13.7	23.8

#### 3.3.3 Highlighting SAs in statin drugs related to DIR risk

Statins, also known as 3-hydroxy-3-methylglutaryl-coenzyme A (HMG-CoA) reductase inhibitors, are commonly used to lower cholesterol and treat cardiovascular disease. However, statin-induced rhabdomyolysis has been widely reported. For example, cerivastatin was withdrawn from the U.S. market because of 52 deaths attributed to rhabdomyolysis and subsequent kidney failure [43]. Therefore, we conducted an in-depth analysis of three statins (cerivastatin, lovastatin, and simvastatin).

As shown in Figs. 7C−E, the structure highlighted in orange in cerivastatin (C) was essentially identical to that in lovastatin (D) and simvastatin (E), representing a common structure shared by statins [44]. Our model's attention map patterns for this structure were nearly identical. Additionally, this structure in cerivastatin showed slight differences from the other two, both structurally and in the attention map, indicating that the model can capture this subtle variation in structure. The structure highlighted in red in lovastatin and simvastatin showed pronounced differences from cerivastatin. Moreover, the regions highlighted in yellow in lovastatin and simvastatin were very similar, with minor variation likely due to an additional methyl group in simvastatin.

We conducted a search for these three drugs on ToxAlerts [45]. For cerivastatin, an aryl halide with an endpoint of acute aquatic toxicity was identified and highlighted in yellow. For lovastatin and simvastatin, carboxylic acid esters with an endpoint of reactive, unstable, or toxic were identified and highlighted in yellow.

#### 3.3.4 Comparing highlighted SAs in drugs associated with DILI risk

Some drugs have very similar structures but differ significantly in properties. One might be relatively safe, while another has been withdrawn due to safety concerns. Given this context, we investigated the relationship between ADRs and molecular structures by analyzing the attention maps of selected drugs related to liver injury.

Ibuprofen is a widely used over-the-counter (OTC) drug prescribed to relieve pain, fever, and inflammation. It has been available on the market for decades and has shown minimal hepatotoxicity. Ibufenac, a structurally similar drug to ibuprofen, was initially effective in treating rheumatoid arthritis. However, ibufenac, introduced to the market in 1966, was withdrawn in 1968 due to severe hepatotoxicity [46].

As shown in Figs. 7F and G, the only structural difference between ibufenac and ibuprofen is the additional methyl group in ibuprofen. Our model clearly identified and focused on this structural variation, which significantly impacts molecular properties.

Zolpidem, widely prescribed for sleep disorders and insomnia since 1992, has shown minimal hepatotoxicity. Alpidem, a structurally similar drug to zolpidem, was initially used to treat anxiety disorders. However, it was withdrawn due to severe hepatotoxicity [46].

As shown in Figs. 7H and I, alpidem and zolpidem differ structurally in both their skeleton and the substituents on the aromatic rings. Our model clearly highlighted the structural differences within their skeleton. Alpidem introduces changes to the substituents on the aromatic ring compared to zolpidem, particularly replacing the methyl groups with chlorine atoms. However, our model placed only slightly emphasis on these substituent changes.

## 4 Discussion

To improve the performance of predicting ADRs and provide comprehensive explainability linking ADRs to molecular structures, we proposed ToxBERT, a model based on the transformer architecture that leverages attention and masking mechanisms to capture structural features associated with specific ADRs using SMILES representations. ToxBERT achieved the highest AUROC across three post-marketing surveillance datasets, outperforming previous QSAR models and peer chemical language models such as MolFormer. Furthermore, ToxBERT significantly enhanced interpretability by highlighting SAs closely associated with ADRs. These capabilities not only aid in uncovering the mechanisms underlying ADRs but also support the proactive identification of potential ADR risks during drug design.

Our model achieved the highest AUROC on three curated datasets and exhibited low standard deviations across metrics, demonstrating its effectiveness in capturing drug features and its robustness. The influences of hyperparameters were analyzed. Masking ratios below 20% or above 80% led to rapid deterioration of performance. We hypothesize that many SMILES tokens (e.g., brackets, numbers, and bonds) serve as auxiliary representations and lack independent meaning. Therefore, with extremely high or low masking ratios, the model either lacks sufficient context or fails to focus on relevant areas, leading to significantly lower AUROC. Additionally, dropout ratios exceeding 50% negatively impacted performance. The randomness introduced by dropout undermined the model's ability to fit the distribution, further hindering its capacity to distinguish between different distributions.

ToxBERT exhibited varying performance in predicting ADRs across different therapeutic categories, showing certain category-specific biases. As shown in Fig. 5, ToxBERT demonstrated lower recall rates for antineoplastic and immunomodulating agents (L) in predicting DIQT and DILI, with rates of 57.7% and 69.6%, respectively. In contrast, the model performed better on drugs in the nervous system (N), with recall rates of 77.8% and 88.9% for DIQT and DILI. It may be partly due to the higher prevalence of these drug categories in the training dataset (for DIQT: N 26.8%, L 18.8%; for DILI: N 17.5%, L 12.0%), which likely enabled the model to effectively learn the structural features of these categories. The model also performed well on drugs in the cardiovascular system (C), achieving a recall rate of 82.2%. Another potential reason is that drugs in the C and N categories are often used as monotherapy for specific diseases, allowing the model to identify clearer relationships between structures and ADRs. Conversely, drugs in the L category are frequently administered in combination therapies, where ADRs arise from complex interactions, making accurate prediction more challenging.

Through the attention mechanism, ToxBERT can effectively identify substructures in drugs associated with ADRs. For the DIQT drugs chloroquine and hydroxychloroquine, as well as DILI drugs ibufenac, ibuprofen, alpidem and zolpidem, ToxBERT not only accurately recognized drug-induced ADR risks but also highlighted associated substructures. For statins in the DIRA dataset, ToxBERT successfully identified the common structures shared among these drugs. Although the link between these structures and toxicity remains unexplored, current studies indicate that lipophilic statins may have a higher risk of muscle toxicity. This is partly attributed to their association with reduced coenzyme Q10 levels, a potential contributor to rhabdomyolysis [47,48]. The identified structures may be related to these mechanisms and their potential toxicity could be validated in future experimental studies.

In this study, we developed ToxBERT, which can assist in predicting the clinical risks of ADRs for marketed drugs and improving drug safety. Additionally, ToxBERT can help exclude high-ADR-risk candidates during the drug development process to reduce the likelihood of clinical trial failures by accurately predicting ADR risks. Furthermore, analyzing the structural features identified by ToxBERT can provide guidance for structural optimization. For instance, it can identify specific substructures associated with ADR risks, which can aid in the design of safer drugs.

Since the model was not trained with information from negative samples, its ability to predict negative samples requires improvement. In future work, it will be necessary to identify false negatives more carefully among samples with low predicted risk values, particularly for drugs with novel structures. Negative predictions can serve as auxiliary information but must be experimentally validated. Future research should explore incorporating features of negative samples into models (such as ensemble learning) to enhance their reliability in predicting negative samples would be explored in future work. Currently, only the chemical structures of the drugs are considered, though drug-induced ADRs involve complex mechanisms. A comprehensive model that incorporates additional factors, such as dosage, administration route, and genomics, could achieve more accurate predictions under complex clinical conditions.

## 5 Conclusion

In conclusion, our study underscores the potential of deep chemical language models for predicting ADRs induced by marketed drugs. ToxBERT outperforms existing approaches across multiple datasets and was validated with external data from FAERS, demonstrating its effectiveness in ADR prediction through enhanced structural representation. Furthermore, the model's ability to identify SAs closely associated with ADRs significantly improves its interpretability and practical applicability. These findings highlight the significance of deep chemical language models, not only in pharmacovigilance but also in facilitating early ADR detection during drug development.

## CRediT authorship contribution statement

**Yujie He:** Investigation, Writing – original draft, Methodology. **Xiang Lv:** Writing – review & editing. **Wulin Long:** Methodology. **Shengqiu Zhai:** Investigation. **Menglong Li:** Supervision, Resources, Funding acquisition. **Zhining Wen:** Writing – review & editing, Methodology, Investigation, Conceptualization.

## Declaration of competing interest

The authors declare that there are no conflicts of interest.
