# Supplementary material for: ToxBERT: an explainable AI framework for enhancing prediction of adverse drug reactions and structural insights
Source: J Pharm Anal. 2025 Jul 3;15(8):101387. doi: 10.1016/j.jpha.2025.101387 (PMC12446765; doi:10.1016/j.jpha.2025.101387)
Supplement: Multimedia component 1 [file mmc1.docx]

# Supplementary Tables

**Table S1** Distribution of drugs across anatomical therapeutic chemical (ATC) codes.

| Contents | ATC Code | DIQT | DIR | DILI |
| --- | --- | --- | --- | --- |
| Alimentary tract and metabolism | A | 38 | 46 | 11 |
| Blood and blood forming organs | B | 21 | 12 | 4 |
| Cardiovascular system | C | 45 | 40 | 3 |
| Dermatologicals | D | 25 | 31 | 12 |
| Genito-urinary system and sex hormones | G | 28 | 29 | 5 |
| Systemic hormonal preparations, excluding  sex hormones and insulins | H | 7 | 12 | 3 |
| Antiinfectives for systemic use | J | 33 | 37 | 11 |
| Antineoplastic and immunomodulating agents | L | 71 | 73 | 23 |
| Musculo-skeletal system | M | 16 | 12 | 2 |
| Nervous system | N | 54 | 51 | 9 |
| Antiparasitic products, insecticides and  repellents | P | 6 | 7 | 2 |
| Respiratory system | R | 16 | 15 | 3 |
| Sensory organs | S | 27 | 27 | 9 |
| Various | V | 18 | 12 | 10 |

DIQT: drug-induced QT prolongation; DIR: drug-induced rhabdomyolysis; DILI: drug-induced liver injury; A: alimentary tract and metabolism; B: blood and blood forming organs; C: cardiovascular system; D: dermatologicals; G: genito-urinary system and sex hormones; H: systemic hormonal preparations, excluding sex hormones and insulins; J: antiinfectives for systemic use; L: antineoplastic and immunomodulating agents; M: musculo-skeletal system; N: nervous system; P: antiparasitic products, insecticides and repellents; R: respiratory system; S: sensory organs;V: various.

# Section S1 Vocabulary Table Construction

The vocabulary table plays a crucial role in language models, including chemical language models, by mapping SMILES strings to numerical representations which are readable for computers. We found that an excessively large vocabulary table not only increases computational cost but also makes the model more challenging to train. MolFormer’s vocabulary table contains many tokens that do not appear in canonical SMILES, such as [237U] and [C@@], which were subsequently removed. To further reduce computational cost and enhance training efficiency, we streamlined the vocabulary table by excluding less common tokens like [W] and [Ir].

# Section S2 Impact of Scaffold Splitting

In training our model, we employed random splitting to partition the dataset. However, randomly dividing drugs may lead to biased performance estimates, as many drugs belong to the same structural class. These drugs often act through similar mechanisms and thus exhibit similar therapeutic effects and adverse drug reactions (ADRs). Consequently, including one drug from a given class in the training set and another from the same class in the test set may cause data leakage, leading to an overestimation of the model’s performance.

To address this potential issue, we explored scaffold splitting, which groups molecules based on their Bemis-Murcko scaffold representations. This approach ensures that structurally similar drugs are consistently assigned to the same subset, providing a reliable validation of the model’s ability across structurally distinct molecules.


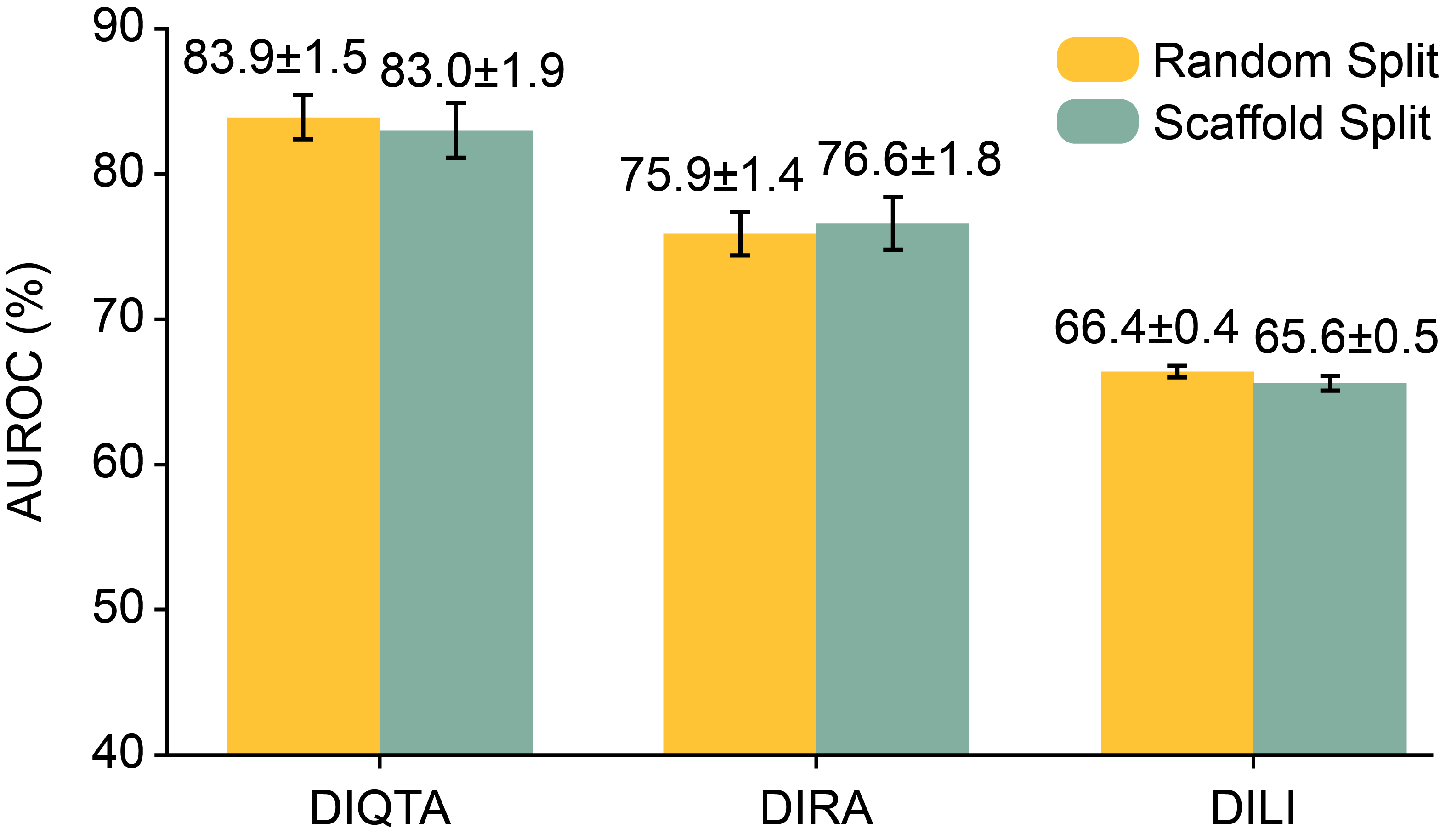


**Fig. S1.** Comparison of the area under the receiver operating characteristic curve (AUROC) between random and scaffold splitting across datasets. The *Y*-axis represents AUROC achieved by models. The *X*-axis represents the various datasets. DIQTA: drug-induced QT prolongation atlas; DIRA: drug-induced rhabdomyolysis atlas; DILI: drug-induced liver injury.

As shown in Fig. [S1,](#_bookmark56) employing scaffold splitting resulted in the area under the receiver operating characteristic curve (AUROC) values of 83.0%, 76.6%, and 65.6% for the DIQTA, DIRA, and DILI datasets, respectively, compared to 83.9%, 75.9%, and 66.4% with random splitting. Performance differences between random and scaffold splitting were minimal.

Additionally, the methods compared in this study also employed random splitting, and scaffold splitting groups molecules based on the Bemis-Murcko scaffold representations, which is a predefined rule. Therefore, we opted for random splitting in this study. Notably, as the dataset size grows, the structural diversity of molecules is also likely to increase. In such cases, scaffold splitting could become more impactful, requiring a more careful evaluation and selection of splitting strategies.

# Section S3 Exploring Performance Variations with Hyperparameters

Based on our model and task design, we aim to leverage certain aspects of overfitting to some extent. Therefore, we conducted experimental investigations into techniques in DL that are highly related to overfitting.


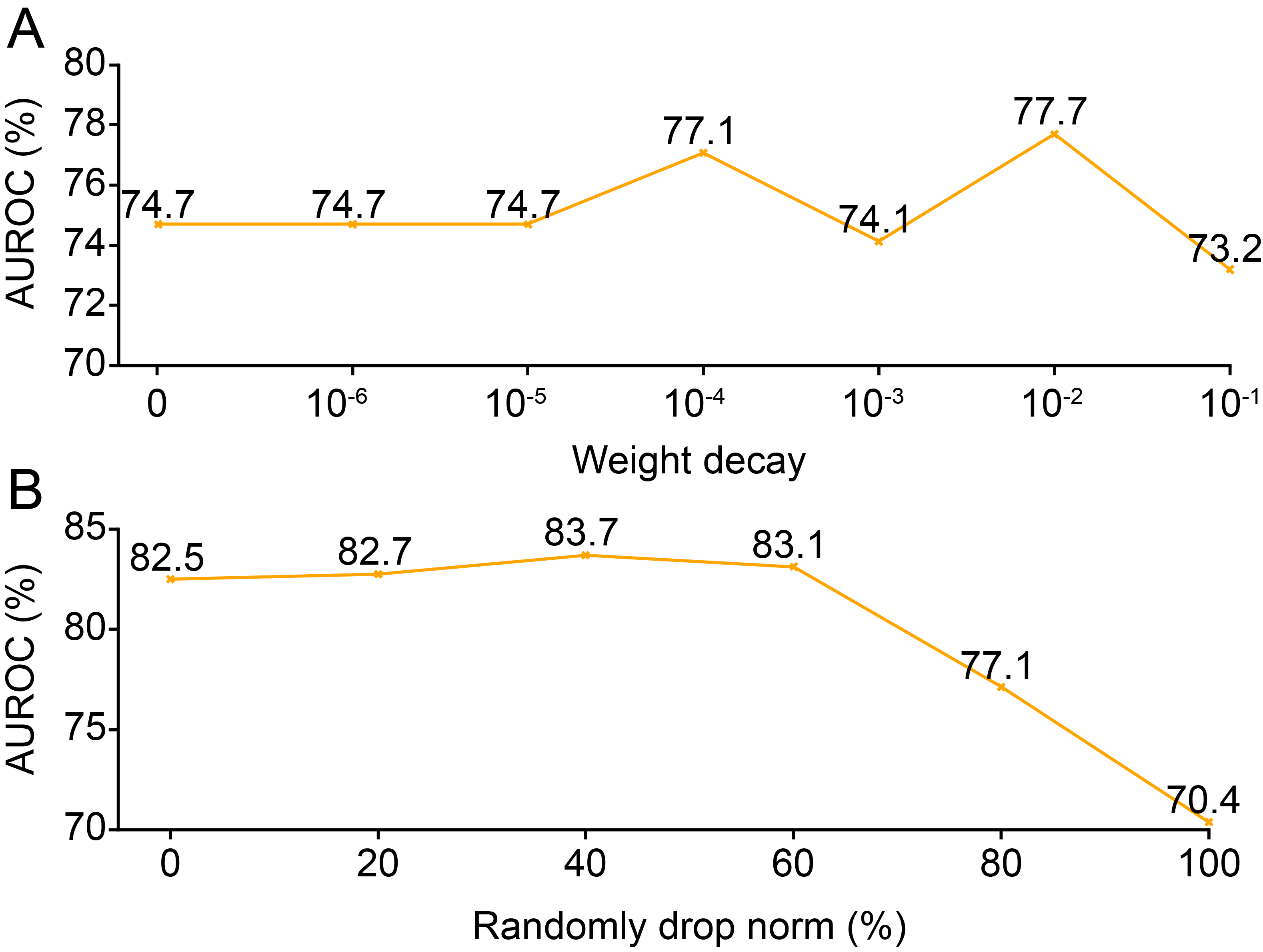


**Fig. S2.** Variations of model performance influenced by overfitting technologies. (A) The impact of weight decay on model performance. The *Y*-axis represents the area under the receiver operating characteristic curve (AUROC) achieved by models. The *X*-axis represents the various weight decay used for training the models. (B) The impact of randomly drop normalization on model performance. The *Y*-axis represents the AUROC achieved by models. The *X*-axis represents the various dropout ratio used for training the models.

*Section S3.1. Weight decay*

In this work, only L2 regularization was used and explored. L2 regularization is a widely employed technique that penalizes the squared magnitude of all parameters directly in the loss function. This prevents excessively large parameter values and encourages smaller, more evenly distributed parameters, allowing the model to utilize features across all dimensions rather than relying heavily on just a few dimensions.

Fig. [S2A](#_bookmark57) shows that L2 regularization had a minimal impact on our model. We believe this is because L2 regularization primarily prevents large parameter values, which does not significantly influence how our model fits the data distribution. However, applying regularization techniques appropriately can still improve the stability and performance of the model.

*Section S3.2. Normalization*

With the forward process of the model, the feature distribution of the data continuously changes. Normalization standardizes the data’s mean to 0 and variance to 1, which ensures the stability of the feature distribution. It also reduces the issue of vanishing gradients. Layer Normalization is commonly used for sequence data, normalizing across all features for each sample.

We randomly removed some Layer Normalization layers to investigate the relationship between model performance and normalization. As shown in Fig. [S2B,](#_bookmark57) the absence of some normalization layers can slightly improve the model’s performance. This may be because normalization alters the data distribution, potentially affecting the model’s ability to learn from it. The original data distribution might allow the model to learn more effectively. However, we also observed during training that the lack of normalization makes the model more difficult to train and converge. Notably, without normalization, the model tends to become highly unstable, making it impossible to achieve proper convergence.

*Section S3.3. Model Architecture*

The number of parameters in a model is often considered a crucial factor influencing overfitting. Even though we aim to leverage aspects of overfitting, excessively large models can still be detrimental. In practice, having a larger model with more parameters is not always better. It is essential to make trade-offs according to the specific requirements of the task.

As shown in the results, our model can be flexibly designed. However, since our model involves the interplay between the Generator and Discriminator, it faces challenges similar to those encountered in GANs. While the performance may improve when both the Generator and Discriminator are more complex, this also makes the model more difficult to train.


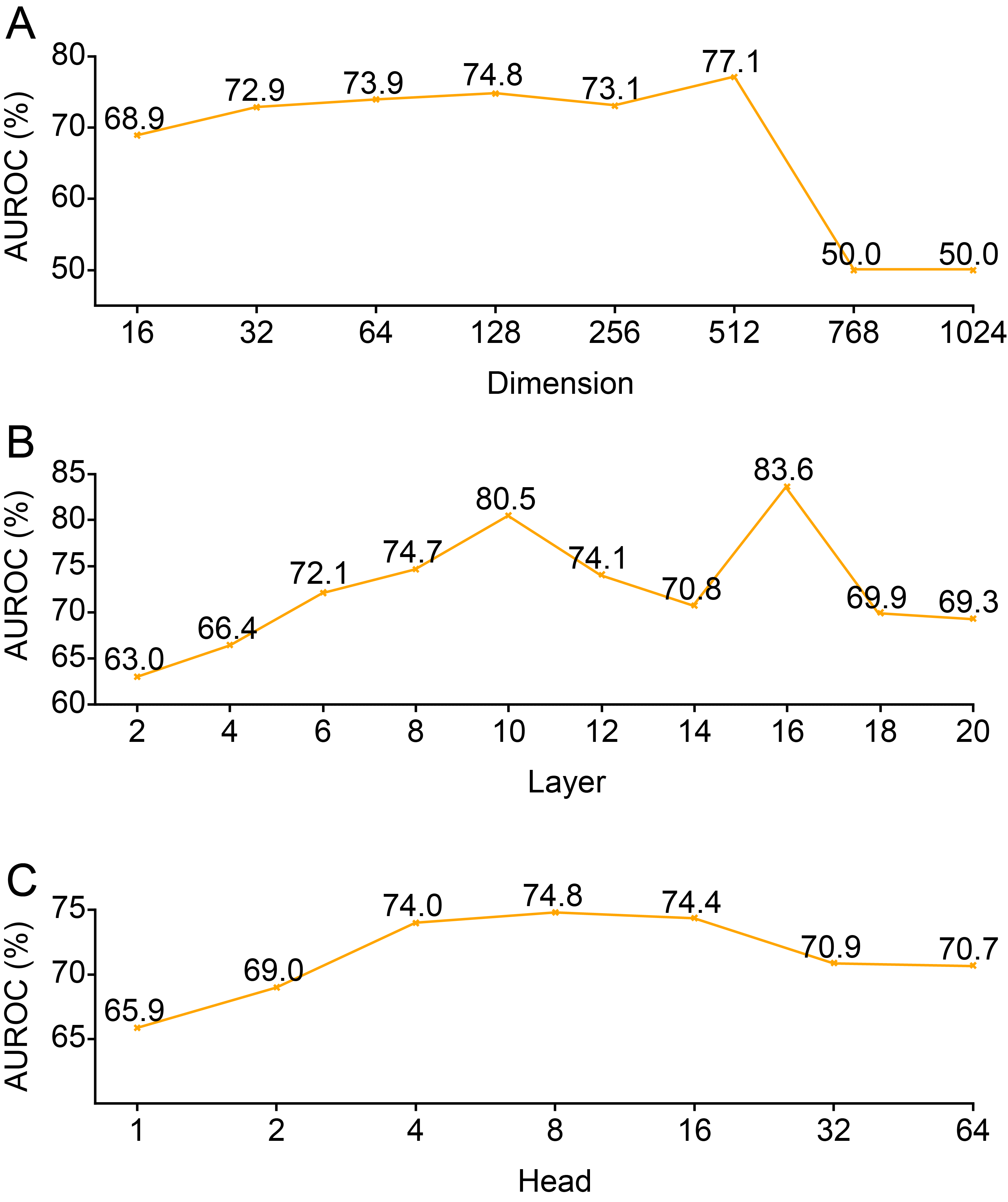


**Fig. S3.** Variations of model performance influenced by model architecture. (A) The impact of dimensions on model performance. The *Y*-axis represents the area under the receiver operating characteristic curve (AUROC) achieved by models. The *X*-axis represents the various dimensions used for training the models. (B) The impact of layers on model performance. The *Y*-axis represents the AUROC achieved by models. The *X*-axis represents the various layers used for training the models. (C) The impact of heads on model performance. The *Y*-axis represents the AUROC achieved by models. The *X*-axis represents the various heads used for training the models.

*Section S3.3.1. Dimension*

As shown in Fig. [S3A,](#_bookmark58) the model’s performance is poor when the dimension is small, due to its limited representation capacity. As the dimension increases, performance first improves and then degrades. Notably, when using an inappropriate learning rate, models with high dimensions can become very unstable, making them difficult to train. Nevertheless, we found that with an appropriate learning rate and a sufficient number of epochs, a larger dimension can result in better performance.

Dimension has a significant impact on the complexity of the model. Given that our vocabulary table is relatively small, it may be challenging to support training with very large dimensions. While larger dimensions enhance the model’s expressiveness and fitting capacity, which aligns with our requirements, they also demand greater computational resources, extended training time, and complicated optimization. Therefore, selecting an appropriate dimension is crucial.

*Section S3.3.2. Layer*

As shown in Fig. [S3B,](#_bookmark58) the model’s performance is poor when the number of layers is small, due to its limited representation capacity. As the number of layers increases, performance first improves and then degrades. Notably, when using an inappropriate learning rate, models with many layers can become very unstable, making them difficult to train. However, we also found that with an appropriate learning rate and a sufficient number of epochs, a larger number of layers can result in better performance.

Similar to dimension, the number of layers has a significant impact on model complexity. Although models with additional layers provide enhanced expressiveness and fitting capacity, they also require increased computational resources and present greater challenges during training. Moreover, smaller models with fewer layers can still achieve satisfactory fitting capabilities. Therefore, selecting the appropriate number of layers should be carefully tailored to the specific task.

*Section S3.3.3. Head*

Multi-head attention is a combination of multiple self-attentions, capturing information from different subspaces of the data simultaneously and then integrating it. It is implemented through transpose and reshape operations.

Fig. [S3C](#_bookmark58) shows that the number of heads has a limited impact on performance. Considering the implementation of multi-head attention, it doesn’t introduce new parameters but simply splits the dimensions. Therefore, it is reasonable that both excessively large and small numbers of heads lead to suboptimal performance.

If the number of heads is too small, the model can only capture limited aspects of features, leading to a decline in performance. Conversely, if it is too large, the dimension assigned to each head becomes too small, reducing the effectiveness of each. The number of heads should be selected based on the embedded dimension and the tasks to achieve optimal results.

# Section S4 Confounding Factors for External Validation

The proportional reporting ratio (PRR) and reporting odds ratio (ROR) are disproportionality analysis (DPA) methods that focus on differences in the proportion of ADR reports. We assume that high PRR scores indicate a strong likelihood that a given drug causes a particular ADR. However, various factors may significantly influence PRR scores for some drug and ADR pairs. Therefore, we propose a further analysis of it.

$$\begin{aligned} \begin{matrix} \mathrm{PRR} & =\frac{N_{TT}/N_{AT}}{N_{TA}/N_{AA}} \end{matrix}\#\left( AUTONUM \backslash* Arabic \right) \end{aligned}$$

Where $N_{TT}$ represents the number of reports of the target ADR for the target drug, and $N_{AT}$ represents the total number of ADRs reported for the target drug. Similarly, $N_{TA}$ represents the number of reports of the target ADR for all other drugs, and $N_{AA}$ represents the total number of ADRs reported for all other drugs.

$$\begin{aligned} \begin{matrix} ROR & =\frac{N_{TT}/N_{OT}}{N_{TO}/N_{OO}} \end{matrix}\#\left( AUTONUM \backslash* Arabic \right) \end{aligned}$$

Where $N_{TT}$ represents the number of reports of the target ADR for the target drug, $N_{OT}$ represents the number of reports of the target ADR for all other drugs, $N_{TO}$ represents the number of reports of all other ADRs for the target drug, and $N_{OO}$ represents the number of reports of all other ADRs for all other drugs.

$$\begin{aligned} log\left( odds \right)=\beta_{0}+\beta_{1}A+\beta_{2}G+\beta_{3}D\#\left( AUTONUM \backslash* Arabic \right) \end{aligned}$$

Where $A$ is age, $G$ is gender, and $D$ indicates whether the drug was administered.

We employed logistic regression to adjust the ROR for age and gender. Adjusted ROR values of the top 10 drugs for each ADR were calculated. As shown in Table [S2,](#_bookmark62) the adjusted ROR values for all these drugs are significantly greater than 1, indicating strong associations between the drugs and their respective ADRs.

For drugs with high PRR values, both PRR and adjusted ROR provided similar insights. Moreover, the results indicate that selecting the top 10 drugs based on PRR is reliable and that our model performs reliably in predicting highly associated drugs without overestimating the model's performance.

**Table S2** Disproportionality analysis for top 10 drugs of three adverse drug reactions in U.S. Food and Drug Administration (FDA) Adverse Event Reporting System (FAERS) dataset.

| Adverse drug reactions | Generic/proper name(s) | PRR | ROR | Adjusted ROR |
| --- | --- | --- | --- | --- |
| DIQT | lactulose | 14.262 | 14.392 | 22.406 |
|  | aminocaproic acid | 12.526 | 12.625 | 20.049 |
|  | felbamate | 12.526 | 12.625 | 20.049 |
|  | ripretinib | 10.220 | 10.284 | 13.947 |
|  | imatinib | 10.214 | 10.278 | 3.833 |
|  | asciminib | 10.214 | 10.278 | 3.833 |
|  | omacetaxine mepesuccinate | 9.875 | 9.935 | 11.650 |
|  | edaravone | 9.122 | 9.172 | 13.702 |
|  | isocarboxazid | 6.699 | 6.725 | 9.986 |
|  | amphotericin b | 6.150 | 6.172 | 7.811 |
| DIR | betaine | 51.297 | 53.433 | 50.510 |
|  | maribavir | 17.265 | 17.490 | 16.600 |
|  | trifluoperazine | 11.266 | 11.359 | 11.870 |
|  | dexamethasone | 11.166 | 11.256 | 13.926 |
|  | valproic acid | 10.122 | 10.196 | 8.350 |
|  | pralatrexate | 9.855 | 9.925 | 12.736 |
|  | quazepam | 9.849 | 9.919 | 11.191 |
|  | sugammadex | 8.089 | 8.134 | 8.892 |
|  | ibuprofen | 8.089 | 8.134 | 8.892 |
|  | rocuronium | 8.089 | 8.134 | 8.892 |
| DILI | vancomycin | 6.188 | 6.202 | 19.090 |
|  | lacosamide | 6.188 | 6.202 | 19.090 |
|  | isosulfan blue | 4.763 | 4.771 | 5.859 |
|  | semaglutide | 4.442 | 4.449 | 8.268 |
|  | abrocitinib | 4.309 | 4.316 | 7.604 |
|  | maribavir | 3.807 | 3.812 | 8.048 |
|  | prednicarbate | 3.702 | 3.706 | 9.967 |
|  | crotamiton | 3.690 | 3.694 | 10.666 |
|  | desonide | 3.482 | 3.486 | 6.187 |
|  | sofosbuvir | 3.444 | 3.447 | 4.657 |

PRR: proportional reporting ratio; ROR: reporting odds ratio; DIQT: drug-induced QT prolongation; DIR: drug-induced rhabdomyolysis; DILI: drug-induced liver injury.
